# Comparison of the Antiviral Activity of Remdesivir, Chloroquine, and Interferon-β as Single or Dual Agents Against the Human Beta-Coronavirus OC43

**DOI:** 10.1089/jir.2022.0210

**Published:** 2023-01-17

**Authors:** Brady T. Hickerson, Faruk Sheikh, Raymond P. Donnelly, Natalia A. Ilyushina

**Affiliations:** Division of Biotechnology Review and Research II, Center for Drug Evaluation and Research, U.S. Food and Drug Administration, Silver Spring, Maryland, USA.

**Keywords:** human coronavirus, pandemic, antiviral, remdesivir, chloroquine, interferon-β, interferon-λ, bronchial epithelial cells

## Abstract

The human beta-coronavirus strain, OC43, provides a useful model for testing the antiviral activity of various agents. We compared the activity of several antiviral drugs against OC43, including remdesivir, chloroquine, interferon (IFN)-β, IFN-λ1, and IFN-λ4, in two distinct cell types: human colorectal carcinoma cell line (HCT-8 cells) and normal human bronchial epithelial (NHBE) cells. We also tested whether these agents mediate additive, synergistic, or antagonistic activity against OC43 infection when used in combination. When used as single agents, remdesivir exhibited stronger antiviral activity than chloroquine, and IFN-β exhibited stronger activity than IFN-λ1 or IFN-λ4 against OC43 in both HCT-8 and NHBE cells. Anakinra (IL-1 inhibitor) and tocilizumab (IL-6 inhibitor) did not mediate any antiviral activity. The combination of IFN-β plus chloroquine or remdesivir resulted in higher synergy scores and higher expression of IFN-stimulated genes than did IFN-β alone. In contrast, the combination of remdesivir plus chloroquine resulted in an antagonistic interaction in NHBE cells. Our findings indicate that the combined use of IFN-β plus remdesivir or chloroquine induces maximal antiviral activity against human coronavirus strain OC43 in primary human respiratory epithelial cells. Furthermore, our experimental OC43 virus infection model provides an excellent method for evaluating the biological activity of antiviral drugs.

## Introduction

Seven, distinct, human coronaviruses (HCoVs) have been identified, four endemic HCoV strains: 229E, OC43, HKU1, and NL63, and three emergent HCoV stains: severe acute respiratory syndrome coronavirus (SARS-CoV), Middle East respiratory syndrome coronavirus (MERS-CoV), and SARS-CoV-2. HCoV OC43 is the most prevalent among the endemic HCoV strains and is responsible for ∼10% of all respiratory tract infections (Nickbakhsh et al, 2020). Outbreaks of OC43 infection occur primarily during winter, and infection typically presents as a common cold but can progress to bronchitis, bronchiolitis, and pneumonia (Choi et al, 2021). Children and elderly individuals are at higher risk of developing severe disease caused by OC43 infection (Lee and Storch, 2014; Patrick et al, 2006). In addition, OC43 was shown to be neuroinvasive in mice and a small subset of patients developed neurological disease during infection (Jacomy and Talbot, 2003; Kasereka and Hawkes, 2021). Currently, no antiviral drugs are approved for treatment of OC43 infection.

The global pandemic caused by SARS-CoV-2 renewed interest in the development of antiviral drugs against HCoV infection. Chloroquine, a widely used antimalarial and autoimmune disease drug, was reported to be effective in inhibiting OC43 and other coronaviruses (Kono et al, 2008; Vincent et al, 2005; Wang et al, 2020). Remdesivir, a nucleoside analog, which inhibits the RNA-dependent RNA polymerase, demonstrated activity against endemic and highly virulent HCoVs (Hsu et al, 2021; Wang et al, 2020). Type I interferons (IFN-α and IFN-β1) and type III interferons (IFN-λ1 and IFN-λ3) were recently shown to inhibit SARS-CoV-2 infection *in vitro* and *in vivo* (Felgenhauer et al, 2020; Mantlo et al, 2020; Vanderheiden et al, 2020). In addition, the risk of patients infected with SARS-CoV-2 to develop a cytokine storm prompted evaluation of several cytokine inhibitory drugs against HCoVs.

Anakinra, a recombinant human IL-1 receptor antagonist, and tocilizumab, a humanized anti-human IL-6 receptor monoclonal antibody, have shown beneficial effects against SARS-CoV-2 infection in patients with COVID-19 (Cavalli et al, 2020). However, the antiviral activity of type I and type III IFNs and cytokine inhibitory drugs, such as anakinra and tocilizumab against OC43 infection, is not well defined.

The OC43 strain is one of the best models for screening anti-HCoV drugs because it belongs to the same genus, *Betacoronavirus*, as the highly virulent betacoronaviruses, SARS-CoV, MERS-CoV, and SARS-CoV-2. Moreover, these four HCoVs have a high level of conservation across their genomes (Lu et al, 2020). However, unlike the highly pathogenic HCoVs, which require biosafety level (BSL)-3 containment, OC43 can be safely used for screening antivirals in a BSL-2 facility. Furthermore, a small animal model of OC43 infection and disease has been developed and used successfully for antiviral trials (Keyaerts et al, 2009). In the present study, we compared the anti-OC43 activities of remdesivir, chloroquine, IFN-β, IFN-λ1, IFN-λ4, anakinra, and tocilizumab in two distinct human epithelial cell lines [i.e., human colorectal carcinoma (HCT-8) and normal human bronchial epithelial (NHBE) cells]. We also tested whether these compounds function additively, synergistically, or antagonistically against OC43 infection when used in combination.

## Materials and Methods

### Cells, viruses, and compounds

HCT-8 (CCL-244) cells were purchased from the American Type Culture Collection (ATCC, Manassas, VA, USA) and maintained in Dulbecco's modified Eagle's medium (DMEM) and 10% fetal bovine serum (FBS). Primary NHBE cells were obtained from Lonza (Walkersville, MD, USA) and were grown on membrane supports at the air–liquid interface as described previously (Matrosovich et al, 2004). Once confluent, the cells were allowed to fully differentiate for at least 3 weeks before experiments. HCoV OC43 (VR-1558) was purchased from ATCC, passaged once on HCT-8 cells in infection medium (DMEM supplemented with 2% FBS) at 33°C and then amplified in HCT-8 cells at 33°C for 6 days to create a working stock. Virus aliquots were stored at −80°C. Remdesivir, anakinra (HY-108841), and tocilizumab (HY-P9917) were obtained from MedChemExpress (Monmouth Junction, NJ, USA). Chloroquine was purchased from eMolecules (San Diego, CA, USA). Human recombinant IFN-β, IFN-λ1, IFN-λ2, IFN-λ3, and IFN-λ4 were obtained from R&D Systems, Inc. (Minneapolis, MN, USA).

Remdesivir and chloroquine were solubilized in 100% dimethyl sulfoxide, and anakinra, tocilizumab, IFN-β, IFN-λ1, and IFN-λ4 were diluted in cell culture medium consisting of RPMI-1640 medium plus 10% FBS. All experimental work was performed in a BSL-2 laboratory approved for use of OC43 by the U.S. Department of Agriculture and the U.S. Centers for Disease Control and Prevention.

### OC43 infectivity assay

The infectivity of OC43 was determined by focus-forming assay in HCT-8 cells and expressed as log_10_ focus-forming units (FFU) per milliliter (Brown et al, 2019). Briefly, confluent monolayers of HCT-8 cells were incubated at 33°C for 1 h with 10-fold serial dilutions of virus. The cells were then washed and overlaid with infection medium containing 1% carboxymethyl cellulose (CMC). After 5 days of incubation at 33°C, the cells were fixed with 10% formalin, permeabilized with 0.1% Triton X-100, and blocked with phosphate-buffered saline (PBS) containing 1% bovine serum albumin and 0.1% Tween-20. OC43 antigen was stained with antibodies [primary: mouse anti-OC43 nucleoprotein antibody (MAB9013; Millipore, Burlington, MA, USA), secondary: goat anti-mouse horseradish peroxidase-conjugated antibody (Sigma-Aldrich, St. Louis, MO, USA)], visualized with SIGMAFast reagent (Sigma-Aldrich), and FFU were visually quantified.

### Antiviral activity assays in HCT-8 and NHBE cells

The antiviral activities of the compounds used alone or in combination were determined by antiviral focus-forming assay in HCT-8 cells and by virus yield reduction assay in NHBE cells. HCT-8 or NHBE cells were pretreated with the antiviral agents at a range of concentrations (remdesivir and chloroquine, 0.0001–100 μM for 2 h; IFN-β, IFN-λ1, and IFN-λ4, 0.0001–1,000 ng/mL for 24 h; anakinra and tocilizumab, 0.1–200 μg/mL for 24 h), which are relevant to the levels obtained *in vivo* (Bartoletti et al, 2022; Brown et al, 2019; Meo et al, 2020; Singh et al, 2020). After pretreatment, cells were infected with OC43 at a multiplicity of infection 0.01 or 1 for HCT-8 or NHBE cells, respectively, diluted in drug-containing infection medium for 1 h at 33°C. After 1 h, virus inoculum was removed, HCT-8 cells were washed once with PBS and overlaid with drug-containing infection medium with 1% CMC. NHBE cells were cultured under air–liquid interface conditions. The supernatants were collected after 48 h and virus yields were determined as the number of FFU/mL in HCT-8 cells.

The drug concentration that caused a 50% decrease in the FFU titer in comparison to control wells without drug was defined as the 50% effective concentration (EC_50_). The results of two to four independent experiments were averaged. Cytotoxicity of all compounds (i.e., CC_50_, the 50% reduction of cell cytotoxicity compared with a cell-only control) was evaluated in HCT-8 and NHBE cells concurrently with each antiviral assay using the CellTiter-Blue Cell Viability Assay (Promega, Madison, WI, USA). Quantification of changes in gene expression was carried out by qRT-PCR analyses of individual IFN-stimulated genes (ISGs) as described previously (Hickerson et al, 2022).

### Western blots

The levels of tyrosine-phosphorylated STAT1 were measured by Western blotting (WB) as described previously (Dickensheets et al, 2013). NHBE cells were treated with recombinant human IFN-λ1, -λ2, or -λ3 (R&D Systems, Inc.) at 500, 50, or 5 ng/mL for 30 min at 37°C. Whole cell lysates were then prepared and assayed directly by WB or after immunoprecipitation of STAT1 protein using a rabbit anti-STAT1 antibody (Santa Cruz Biotechnology, Inc., Santa Cruz, CA, USA). The protein extracts were resolved by electrophoresis on 8% polyacrylamide Tris-Glycine gels (Invitrogen, Carlsbad, CA, USA), and then transferred to polyvinylidene difluoride membranes. The levels of tyrosine-phosphorylated STAT1 were measured by immunoblotting with mouse monoclonal anti-phospho-Y701-STAT1 Ab (Cell Signaling Technology, Beverly, MA, USA). The levels of total STAT1 protein were measured by reprobing the blots with an anti-STAT1 antibody.

## Results

**FIG. 1.**
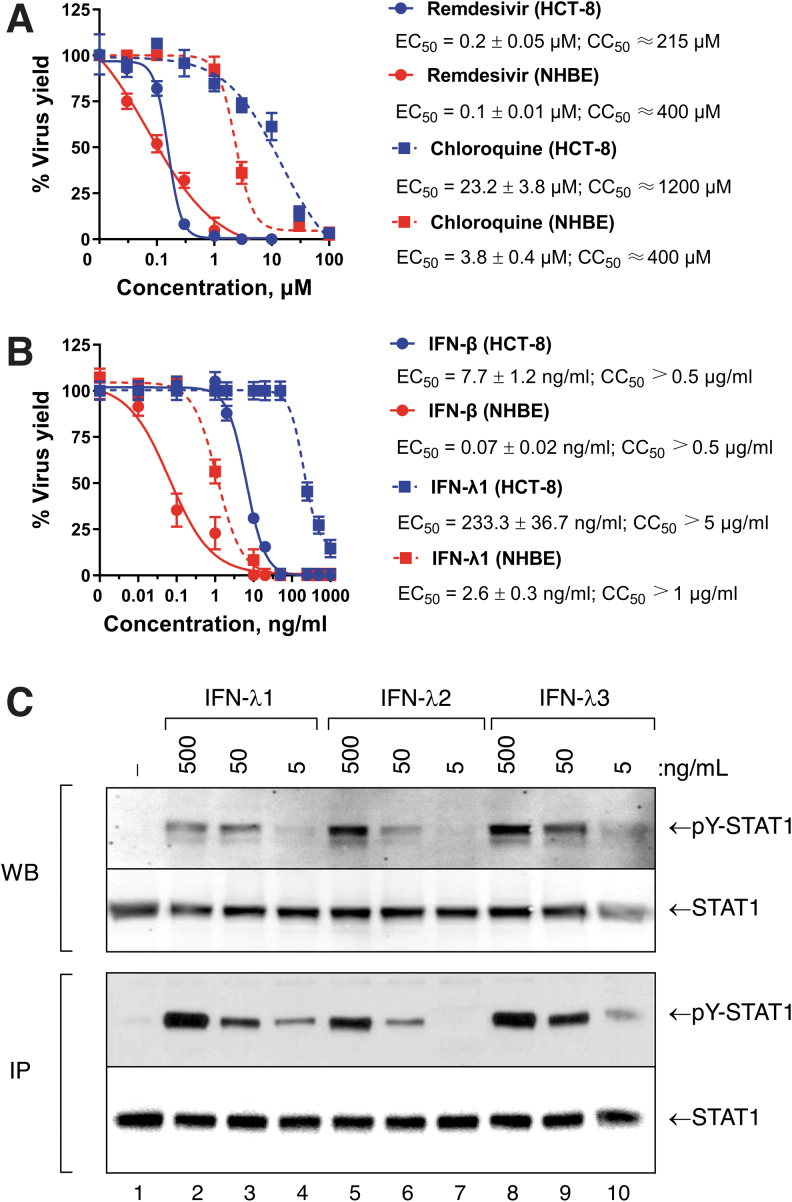
Antiviral activity of **(A)** remdesivir (μM) and chloroquine (μM), and **(B)** IFN-β (ng/mL) and IFN-λ1 (ng/mL) against OC43 in HCT-8 and NHBE cells as measured by focus-forming assay in HCT-8 cells and virus yield reduction assay in NHBE cells. The mean EC_50_ values and standard deviations were determined based on data from at least three independent experiments. **(C)** IFN-λs induce tyrosine-phosphorylation of STAT1 in a dose-dependent manner in NHBE cells. Cultures of NHBE cells were treated with recombinant human IFN-λ1, -λ2, or -λ3 at 500, 50, or 5 ng/mL for 30 min at 37°C. Whole cell lysates were then prepared and assayed directly by WB or after immunoprecipitation of total STAT1 protein using a rabbit anti-STAT1 antibody. The levels of tyrosine-phosphorylated STAT1 and total STAT1 were assayed by WB with anti-pY-STAT1 and anti-STAT1 mAbs, respectively. HCT, human colorectal carcinoma; IFN, interferon; NHBE, normal human bronchial epithelial; WB, Western blotting.

We first tested the sensitivity of OC43 to treatment with remdesivir, chloroquine, IFN-β, IFN-λ1, IFN-λ4, anakinra, or tocilizumab as single agents. The mean EC_50_ values for remdesivir were 0.2 and 0.1 μM in HCT-8 and NHBE cells, respectively (Fig. 1A). In contrast, the sensitivity of OC43 to chloroquine differed significantly in each cell line: 23.2 μM in HCT-8 versus 3.8 μM in NHBE cells. The CC_50_ values obtained for both drugs were ≥215 μM in both cell lines. Furthermore, IFN-β showed strong antiviral activity against OC43 with a mean EC_50_ that ranged from 0.07 to 7.7 ng/mL in NHBE and HCT-8 cells, respectively (CC_50_ values >0.5 μg/mL; Fig. 1B). Relative to IFN-β, IFN-λ1 was ∼28.2-fold less effective (*P* < 0.05) against OC43 virus in both cell lines (CC_50_ values >1 μg/mL). Although the antiviral activity of IFN-λ1 was significantly lower than IFN-β against the OC43 virus (Fig. 1B), we confirmed that the weaker potency of IFN-λ1 was not due to an inability to signal at the concentrations tested. As shown in Figure 1C, treatment of NHBE cells with recombinant human IFN-λ1, -λ2, or -λ3 induced activation of phospho-STAT1 in a dose-dependent manner.

Furthermore, IFN-λ4 also did not inhibit OC43 viral replication at concentrations <1 μg/mL in HCT-8 cells (Table 1). The EC_50_ values for anakinra and tocilizumab ranged from 129.9 to 229.8 μg/mL, respectively, in HCT-8, and these agents did not exhibit antiviral activity against OC43 at concentrations below 100 μg/mL in NHBE cells (Table 1).

**Table 1.** Antiviral Activity of Interferon-λ4, Anakinra, and Tocilizumab Against OC43 *In Vitro*

Compounds	HCT-8 cells		NHBE cells	
	EC_50_	CC_50_	EC_50_	CC_50_
IFN-λ4	>1 μg/mL	>5 μg/mL	779.6 ± 19.0 ng/mL	>1 μg/mL
Anakinra	229.8 ± 81.8 μg/mL	≈100 mg/mL	>100 μg/mL	>100 μg/mL
Tocilizumab	129.9 ± 39.6 μg/mL	>10 mg/mL	>100 μg/mL	>100 μg/mL

EC_50_ values were determined by antiviral focus forming assay after 5 or 2 days postinfection in HCT-8 cells or NHBE cells, respectively. CC_50_ values were determined with serially diluted compounds concurrently with each antiviral assay at 5 or 2 days postincubation in HCT-8 or NHBE cells, respectively, using the CellTiter-Blue Cell Viability Assay (Promega).

CC_50_, 50% reduction of cell cytotoxicity; EC_50_, 50% effective concentration; HCT, human colorectal carcinoma; IFN, interferon; NHBE, normal human bronchial epithelial.

**FIG. 2.**
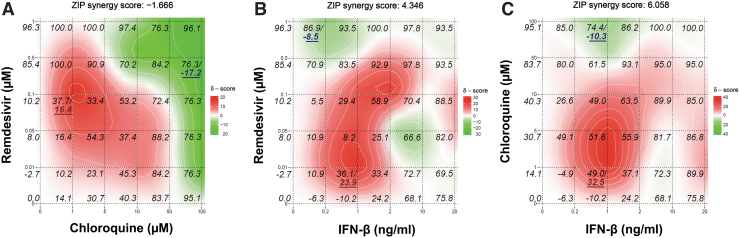
Combinatorial effects of remdesivir plus chloroquine **(A)**, remdesivir plus IFN-β **(B)**, or chloroquine plus IFN-β **(C)** against OC43 in HCT-8 cells. The percentage of viral inhibition together with the highest synergy and lowest antagonism scores are shown in *italics* on the three-dimensional interaction landscapes of antiviral drugs, which were generated by Synergy Finder (Ianevski et al., 2017) based on the ZIP independence model. *Red color* indicates synergy and *green color* indicates antagonism of the two test drugs. ZIP, Zero Interaction Potency.

We next evaluated the antiviral activity of combined treatment with remdesivir, chloroquine, or IFN-β when two of these compounds were used together against OC43 in HCT-8 cells (Fig. 2). We analyzed the dose–response matrix of the virus yield inhibition using the Zero Interaction Potency model (Yadav et al, 2015) using Synergy Finder (Ianevski et al, 2020) and characterized the interaction between the test drugs in each combination treatment. The combined action of IFN-β at 1 ng/mL plus remdesivir at 0.01 μM or chloroquine 1 μM reached a maximum synergy score of 23.9 or 32.5, respectively. In contrast, the combination of remdesivir at 0.5 μM plus chloroquine at 100 μM exhibited a maximum antagonism score of −17.2 in HCT-8 cells (Fig. 2).

**FIG. 3.**
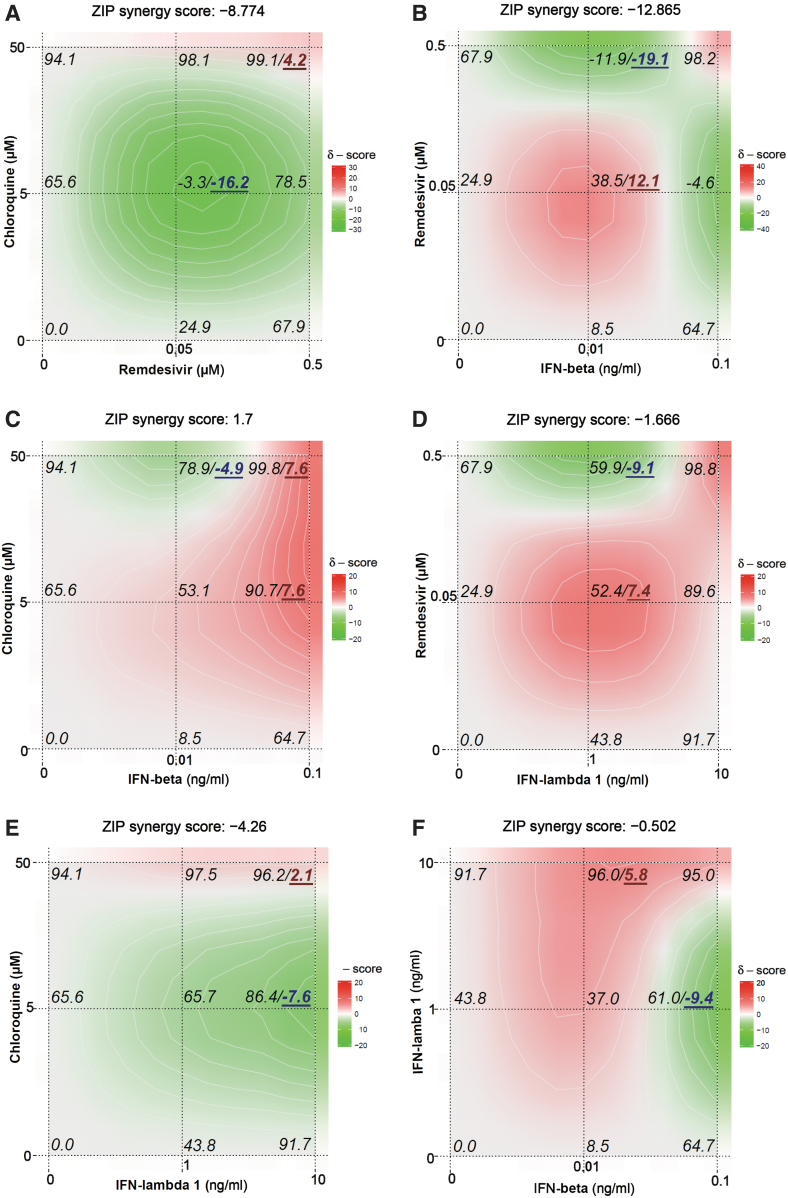
Combinational effect of chloroquine plus remdesivir **(A)**, remdesivir plus IFN-β **(B)**, chloroquine plus IFN-β **(C)**, remdesivir plus IFN-λ1 **(D)**, chloroquine plus IFN-λ1 **(E)**, and IFN-β plus IFN-λ1 **(F)** against OC43 in NHBE cells. The percentage of viral inhibition together with the highest synergy and lowest antagonism scores are shown in *italics* on the three-dimensional interaction landscapes of antiviral drugs, which were generated by Synergy Finder (Ianevski et al., 2017) based on the ZIP independence model. *Red color* indicates synergy and *green color* indicates antagonism of the two drugs.

We further examined the combinational effect of remdesivir, chloroquine, IFN-β, or IFN-λ1 when these agents were combined with each other in NHBE cells (Fig. 3). The overall interaction between chloroquine combined with remdesivir or IFN-λ1 was antagonistic in inhibiting OC43 yield, and the combination of remdesivir (0.05 μM) plus chloroquine (5 μM) demonstrated the highest antagonism score of −16.2. In contrast, the combination of IFN-β plus either remdesivir, chloroquine, or IFN-λ1 was usually synergistic at most concentrations tested, but some regions of additivity or antagonism were also observed. The combined activity of IFN-β at 0.01 ng/mL plus remdesivir at 0.05 μM reached a maximum synergy score of 12.1 among all combinations tested in NHBE cells (Fig. 3).

**FIG. 4.**
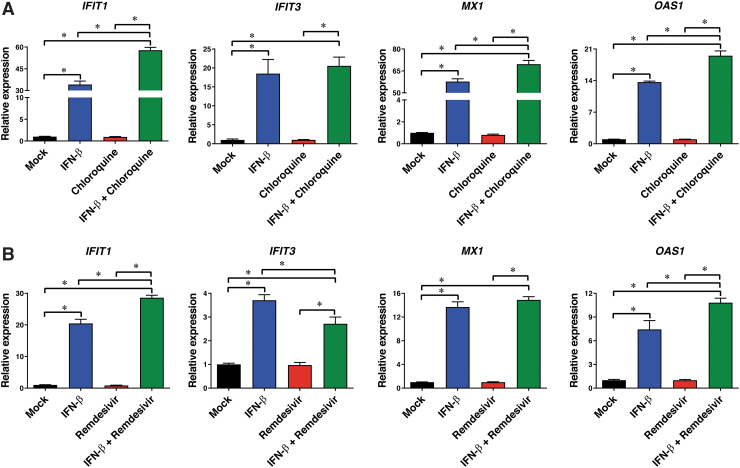
Effects of treatment with IFN-β (1 ng/mL), chloroquine (1 μM), or IFN-β plus chloroquine **(A)** versus treatment with IFN-β (0.01 ng/mL), remdesivir (0.05 μM), or IFN-β plus remdesivir **(B)** on antiviral gene expression (*IFIT1*, *IFIT3*, *MX1*, and *OAS1*) by NHBE cells. *P < 0.05, one-way ANOVA.

We next measured the ability of the optimal drug combinations with the highest synergy scores (i.e., IFN-β at 1 ng/mL combined with chloroquine at 1 μM in HCT-8 cells and IFN-β at 0.01 ng/mL combined with remdesivir at 0.05 μM in NHBE cells) to induce *IFIT1/3*, *MX1*, and *OAS1* expression at 24 h posttreatment in HCT-8 (Fig. 4A) and NHBE (Fig. 4B) cells. Treatment with IFN-β at 1.0 or 0.01 ng/mL upregulated expression of all ISGs (*P* < 0.05). In contrast, chloroquine or remdesivir used as single agents failed to significantly increase the ISG expression levels compared with untreated control cells. However, when chloroquine or remdesivir were combined with IFN-β, *IFIT1*, *OAS1*, and *MX1*, expression levels were significantly increased in HCT-8 and NHBE cells, respectively, compared with the cells treated with IFN-β alone (1.4-fold, *P* < 0.05).

## Discussion

In this study, we found that remdesivir exhibited stronger antiviral activity than chloroquine against the OC43 betacoronavirus. We also found that IFN-β (type I IFN) exhibited stronger antiviral activity than IFN-λ1 or IFN-λ4 (type III IFNs) against OC43 infection in both HCT-8 and NHBE cells. To our knowledge, this is the first report to demonstrate the antiviral effectiveness of type I and type III IFNs against the OC43 virus. The reason why IFN-β induced stronger antiviral activity than IFN-λ1 against OC43 virus is not clear but might reflect differences in the relative levels of their corresponding cell surface receptors, the kinetics of ISG expression, and/or differences in the binding affinities of their cognate receptors.

Previous studies have shown the clinical benefit of administration of certain cytokine inhibitors, including anakinra (IL-1 inhibitor) and tocilizumab (IL-6 inhibitor), in patients with severe COVID-19 (Cavalli et al, 2020; Huet et al, 2020; Somers et al, 2021; Toniati et al, 2020). However, neither of these agents exhibited direct antiviral activity against OC43 infection in our experimental infection model. Therefore, it is likely that the positive clinical effects of anakinra and tocilizumab in patients with severe COVID-19 are due to their ability to block the systemic inflammation induced by IL-1 and IL-6, respectively. In contrast, while chloroquine was able to inhibit OC43 infection in our *in vitro* cell culture model, preclinical studies of hydroxychloroquine in nonhuman primates showed that treatment with this drug is unlikely to have any significant antiviral activity against COVID-19 in humans (Maisonnasse et al, 2020).

The use of two or more antiviral and/or immunomodulatory drugs might induce more effective control of viral infection. In addition, the use of two or more drugs in combination might result in more effective antiviral activity by decreasing the concentration levels of each single drug alone. We found that the combination of drugs with different mechanisms of action (e.g., IFN-β combined with chloroquine or remdesivir) resulted in high synergy scores. Combined treatment with IFN-β plus chloroquine or remdesivir also induced higher levels of ISG expression than did treatment with IFN-β alone. The mechanism by which the combination of IFN-β plus remdesivir or chloroquine resulted in stronger antiviral activity and higher expression of ISGs than that induced by IFN-β alone is unknown. It is possible that either or both of these drugs upregulate type-I IFN receptors and/or their rate of turnover; however, additional studies are needed to definitively address this question.

Our finding that treatment with IFN-β plus remdesivir may be more effective than IFN-β alone as a means to treat betacoronavirus infections correlates well with a previous study, which showed that early cotreatment with IFN-β plus remdesivir was more effective than remdesivir alone in alleviating symptoms and in shortening viral shedding and hospitalization in high-risk COVID-19 patients (Tam et al, 2022).

We observed antagonistic effects of certain combinations of chloroquine and remdesivir against OC43 in NHBE cells. The basis for this unexpected antagonism can be explained, at least in part, by analysis of the raw data, which showed that remdesivir alone reduced the OC43 virus yield to the maximum extent, thereby obscuring any potential additional antiviral effect of chloroquine. Our findings correlate well with a related previous report by others (Bobrowski et al, 2021), which showed that chloroquine also antagonized the antiviral activity of remdesivir against SARS-CoV-2 in a cell culture model.

Our *in vitro* OC43 virus infection assay provides a useful method for safely screening a wide variety of agents for antiviral activity against a model betacoronavirus. We found that NHBE cells were more sensitive to treatment with remdesivir, chloroquine, IFN-β, or IFN-λ1 than HCT-8 cells, and yielded EC_50_ values similar to those observed previously by others (Brown et al, 2019; Keyaerts et al, 2009). Moreover, antagonistic interaction between chloroquine and remdesivir was observed only in NHBE, not in HCT-8 cells. NHBE cell cultures functionally recapitulate human airway epithelium, and they are the primary target cells for OC43 infection *in vivo*. Consequently, NHBE cells provide an excellent *in vitro* model for the initial screening of antiviral drugs against OC43 infection.
